# The ERK-p38MAPK-STAT3 Signalling Axis Regulates iNOS Expression and *Salmonella* Infection in Senescent Cells

**DOI:** 10.3389/fcimb.2021.744013

**Published:** 2021-10-22

**Authors:** Sheryl Erica Fernandes, Deepak Kumar Saini

**Affiliations:** ^1^ Department of Molecular Reproduction, Development and Genetics, Indian Institute of Science, Bangalore, India; ^2^ Center For BioSystems Science and Engineering, Indian Institute of Science, Bangalore, India

**Keywords:** signalling, senescence, Salmonella typhimurium, ERK, p38 MAPK, STAT3, iNOS

## Abstract

The cellular changes occurring due to senescence like proliferation arrest, increase in free radical levels, and secretion of pro-inflammatory cytokines have been well studied, but its associated alteration in intracellular signalling networks has been scarcely explored. In this study, we examine the roles of three major kinases *viz*. p38 MAPK, ERK, and STAT3 in regulating iNOS expression and thereby the levels of the free radical Nitric oxide in senescent cells. Our study revealed that these kinases could differentially regulate iNOS in senescent cells compared to non-senescent cells. Further, we tested the physiological relevance of these alterations with *Salmonella* infection assays and established an inter-regulatory network between these kinases unique to infected senescent cells. Overall, our findings show how key signalling networks may be rewired in senescent cells rendering them phenotypically different.

## Introduction

Senescence is defined as a state of irreversible cell cycle arrest wherein intrinsic permanent changes prevent the cell from undergoing division even in the presence of appropriate mitogenic cues (Hernandez-segura et al., 2018). The quest to identify factors contributing to this divisional arrest led to the discovery of persistent DNA damage as one of the major triggers caused by telomere attrition, oxidative or genotoxic stress (López-Otín et al., 2013; Rossiello et al., 2014; Salama et al., 2014; Hernandez-segura et al., 2018). Following this discovery, several groups have elucidated the role of DNA damage response (DDR) signalling in senescence induction and many proteins involved in the signalling cascade like γH2A.x and p53 binding protein (53BP1) have also emerged as important markers of cellular senescence, in addition to cell cycle checkpoint proteins like p21 and p16 (Chen et al., 2007; Baker and Fruk, 2021). Senescent cells also possess unique features that distinguish them from other non-dividing quiescent or terminally differentiated cells. These include their ability to secrete several factors comprising the Senescence Associated Secretory Phenotype (SASP) that can alter its surrounding micro-environment (Coppé et al., 2008; Coppé et al., 2010), their comparatively higher resistance to apoptosis when insulted by certain stressors (Soto-gamez et al., 2019; Ogrodnik, 2021) and accumulation of free radicals like Reactive Oxygen Species (ROS) and Nitric Oxide (NO) (Davalli et al., 2016; Liguori et al., 2018).

Subsequently, many studies have explained the underpinning signalling changes that regulate the senescence phenotype. For instance, it has been demonstrated that p21 in DNA damage-induced senescent cells promotes cell viability by modulating the activation of JNK and pro-caspases, providing evidence of crosstalk between DNA damage signalling and pro-survival pathways (Yosef et al., 2017). Another study shows how inhibition of p38 MAPK in senescent cells significantly reduces many proinflammatory components of the SASP and its activation is modulated by p53 (Freund et al., 2011). Further, several attempts to understand metabolic reprogramming and resistance to autophagy-induced death in senescent cells have uncovered the pivotal role of mTORC1 in regulating mitochondrial function and unfolded protein response (UPR) (Narita et al., 2011; Correia-melo et al., 2016; Soto-gamez et al., 2019).

In this study, we focus on how senescence-associated altered signalling pathways affect yet another aspect, the host cell response to bacterial infection. It was recently demonstrated that senescent cells have elevated anti-bacterial immune responses. More specifically, higher levels of inducible Nitric Oxide Synthase (iNOS) were responsible for reduced bacterial survival in senescent cells (Fernandes et al., 2021). Additionally, the role of p38 MAPK as a negative regulator of iNOS was implicated, but the underlying molecular mechanism remained elusive. Here, we investigate the roles of p38 MAPK, ERK and STAT3, the major kinases known to regulate immune responses to bacterial infection in non-senescent cells (Hobbie et al., 1997; Yu et al., 2002; Lin et al., 2003; Arbibe et al., 2007; Queval et al., 2016; Panagi et al., 2020) and propose a model of cross-regulation between these signalling pathways that is unique to senescent cells and impinges on its response to infection.

## Materials and Methods

### Cell Culture and Induction of Senescence

HeLa and HepG2 (ATCC, USA) were maintained in DMEM (Sigma Aldrich, USA) supplemented with 10% FBS (Invitrogen). For senescence induction, cells were treated for 48h with 100 µM 5-Bromo-2’-deoxyuridine (Sigma Aldrich, USA).

### *Salmonella* Infection

An overnight culture obtained from an isolated colony of *S*.Typhimurium NCTC 12023 grown on a *Salmonella-Shigella* (SS) agar plate was diluted in Luria Bertani (LB) broth and incubated for 6h at 37°C/180 rpm (log-phase culture-OD_600_ 1.0). The bacterial culture was then washed and resuspended in sterile phosphate-buffered saline (PBS) prior to infection.

To quantify infection, bacteria were added to a monolayer of non-senescent or senescent HeLa/HepG2 cells (MOI 1:10) that were untreated or pre-treated with vehicle (DMSO) or inhibitors for 4h. The cells were centrifuged at 250×g for 10 min (25°C) (to ensure synchronous bacterial invasion) and then incubated for 1h at 37°C in a 5% CO2 humidified atmosphere (invasion). At the end of co-incubation, the medium was replaced with complete medium containing 100 µg/mL Gentamicin for 30 minutes to kill extracellular bacteria. Cells were then lysed in 0.5% Triton X-100 (v/v in PBS) to enumerate the number of invading bacteria by plating lysates on SS agar or maintained in medium supplemented with 10 μg/ml gentamicin until 16h at which they were lysed to determine intracellular bacterial replication. All the inhibitors were purchased from Cayman Chemical Co., USA and the concentration of inhibitors used was as follows: SB 202190-10µM (p38MPAKi), PD 184162-1 µM (ERKi), AG 490-40 µM (STAT3i). Aminoguanidine hemisulfate salt was purchased from Sigma Aldrich, USA and was used at a final concentration of 10µM.

### Gene Expression Analysis

After isolating total RNA using TRI reagent (Sigma, USA), cDNA was synthesized using iScript cDNA Synthesis Kit (Bio-Rad, USA) followed by quantitative expression analysis using SYBR Green qPCR Kit (Thermo Fisher Scientific, USA) as per manufacturer’s instructions. Gene expression levels were normalised to β-actin expression. RotorGene-Q (Qiagen, Germany) real-time instrument and associated software was used for data and melting curves analysis. The primer sequences have been enlisted **Table S1**.

### Western Blotting

Cell were lysed in Mammalian protein extraction buffer (GE Healthcare, USA) as per the manufacturer’s specifications. 60-100µg of total protein was used for analysis. All the primary antibodies were from CST (Cell Signalling Technology Inc., USA), the dilutions used has been specified in **Table S2**. The blots were imaged in ChemiDoc MP Imaging System (Bio-Rad Inc., USA) at multiple exposure settings.

### Estimation of Cellular ROS

Cells were incubated in medium containing 10 µM 2′,7′-dichlorofluorescein (DCFDA) (Sigma, USA) for 30 min in the dark, washed with PBS and then analysed for Dichlorofluorescein fluorescence (Infinite F200, Tecan, Austria) at an excitation wavelength of 492 nm and emission wavelength of 525 nm. Cells were counted to express fluorescence per cell.

## Results

### STAT3 and p38 MAPK Activation Is Altered in Infected Senescent HeLa Cells

Given that p38MAPK, ERK and STAT3 are known to regulate infection in non-senescent cells, we first sought to compare the activation kinetics of these kinases between non-senescent and senescent cells after infection. For this, we adopted the *Salmonella* Typhimurium- senescent cell infection model, which has been described previously and shows reduced bacterial infection in senescent cells (Fernandes et al., 2021). In this study, we have used two host cell lines: HeLa, which is commonly used to study *Salmonella* pathogenesis and host responses to its infection (Takemura et al., 2021; Yang et al., 2021) and HepG2 (Hannemann et al., 2013), a human hepatocyte cell line as the liver is a primary target of the bacterium during the systemic disease, Typhoid (Watson and Holden, 2010). Since persistent DNA damage is the major trigger of cellular senescence, a DNA-damage induced senescent cell model was used. In this model, cells are treated with BrdU, a genotoxic agent which specifically induces only DNA damage and subsequently activates the DDR signalling cascade resulting in proliferation arrest and senescence (Eriko et al., 1999; Minagawa et al., 2005; Rossa et al., 2008; Le et al., 2010; Nair et al., 2014; Nair et al., 2018; Ozsvari et al., 2018). Moreover, this model has also been previously used to study the effects of senescence on *Salmonella* infection (Lim et al., 2010; Fernandes et al., 2021).

**Figure 1:**
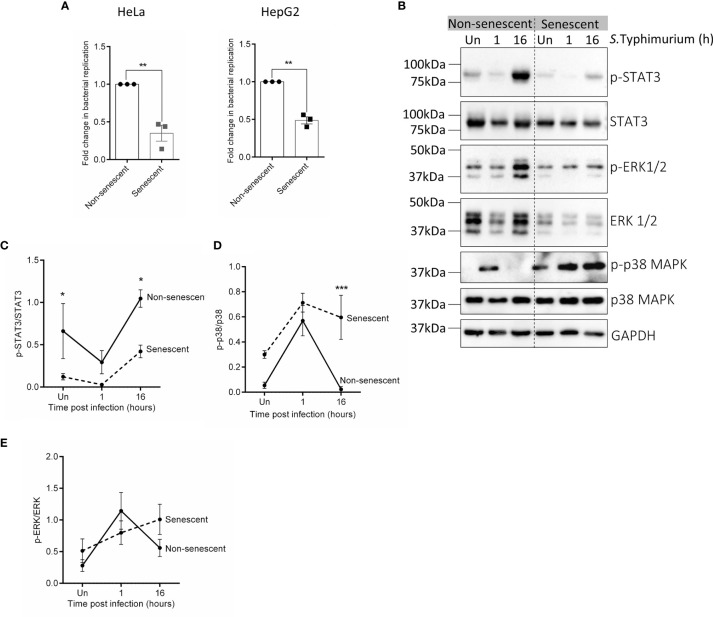
Infection and activation of various kinase varies between non-senescent and senescent HeLa cells. **(A)** Equal numbers of non-senescent and senescent HeLa or HepG2 cells were seeded and co-incubated with S.Typhimurium (MOI=10) for 1h (invasion). Extracellular bacteria were then killed with 100µg/mL Gentamicin containing complete medium for 30 minutes and host cells were then lysed to enumerate the number of bacteria that had invaded. Parallel wells were maintained in 10 µg/mL Gentamicin containing medium for 16h after which host cells were lysed to enumerate viable bacteria in the cells at 16h post-invasion. Bacterial replication was determined by dividing CFU at 16h by CFU at 1h of infection (invasion). Fold change in bacterial replication was then plotted relative to nonsenescent cells. Statistical significance of differences was analysed by Independent t-test, **P ≤ 0.01. **(B)** Western blot for phosphorylated and total levels of STAT3, ERK1/2 and p38 MAPK in uninfected and infected non-senescent and senescent HeLa cells. GAPDH is used as a loading control. **(C–E)** Quantification of phosphorylated levels of STAT3 **(C)**, p38 MAPK **(D)** and ERK1/2 **(E)** relative to their total levels, respectively in non-senescent and senescent HeLa cells. The data represents mean ± SEM from atleast three independent experiments. Statistical significance of differences was analysed by Two-way ANOVA followed by posthoc Fisher’s LSD test, *P ≤ 0.05, ***P ≤ 0.001.

Prior to the infection experiments, senescence in HeLa and HepG2 cells after BrdU treatment was confirmed by determining the expression of *CDKN1a* which is a marker of proliferation arrest in senescent cells (Chen et al., 2007). A higher expression of *CDKN1a* in cells treated with BrdU ascertained that the cells were senescent (**Supplementary Figure S1**). Further, to validate if indeed senescent cells had significantly reduced intracellular bacterial loads, a standard Gentamicin protection assay was performed after infecting equal numbers of non-senescent and senescent HeLa or HepG2 cells with *S*.Typhimurium (Multiplicity of infection, MOI=10). An increase in intracellular bacterial CFU at 16h relative to 1h of infection (referred hereafter to as “bacterial replication”) was compared between non-senescent and senescent cells and has been represented as a fold change relative to non-senescent cells. In both the cell lines, bacterial replication was atleast 2-fold higher in non-senescent cells compared to senescent cells (**Figure 1A**), thus confirming that bacterial survival is significantly compromised in senescent cells.

Next, we examined the activation of the key kinases p38 MAPK, ERK and the final downstream effector target of JAK1/2 pathway i.e., STAT3 in uninfected non-senescent and senescent cells and at 1h and 16h post-infection. We observed that p-STAT3 levels were significantly lower in uninfected senescent HeLa and remained lower than its levels in non-senescent HeLa even after infection (**Figures 1B, C**). However, activation of p38 MAPK was higher in senescent HeLa and its levels remained persistently more elevated than its levels in non-senescent cells during infection (**Figures 1B, D**). We did not observe any change in p-ERK levels between uninfected or infected non-senescent and senescent HeLa cells (**Figures 1B, E**). Since we observed a significant alteration in the activation of p38 MAPK and STAT3 in HeLa, we also compared p-p38 MAPK (**Supplementary Figures S2A, B**) and p-STAT3 levels (**Supplementary Figures S2A, C**) in uninfected and infected non-senescent and senescent HepG2 cells. However, there was no significant difference in their basal phosphorylated levels or even after infection.

### STAT3, p38 MAPK and ERK Regulate iNOS Differently in Senescent Cells Affecting Bacterial Infection

To further understand if these kinases play differential roles during infection in non-senescent and senescent cells, their functions were abrogated using specific molecular inhibitors and bacterial survival in inhibitor-treated cells was compared to the vehicle control. Downstream activity of p38 MAPK was inhibited using SB 202190 (p38MAPKi) (Riis et al., 2011), which reduced phosphorylation of its substrate Hsp27 and ascertained inhibitor activity (**Supplementary Figure S3A**). To inhibit ERK and STAT3 activation, we used molecular inhibitors targeting their upstream kinase activators, MEK using PD-184161 (ERKi) (Cowan and Storey, 2003; Klein et al., 2006) and JAK1/2 using AG490 (STAT3i) (Meydan et al., 1996; Price et al., 2015). Reduced p-ERK and p-STAT3 levels in non-senescent and senescent cells after inhibitor treatment were confirmed by western blotting (**Supplementary Figures S3B, C**). In addition to validating inhibitor activity in the host cells, the absence of a direct effect of these molecules on *Salmonella* viability was also confirmed by growing bacteria in Luria Bertani (LB) broth containing the inhibitors at the same concentration used in the infection experiments (**Supplementary Figure S3D**).

**Figure 2:**
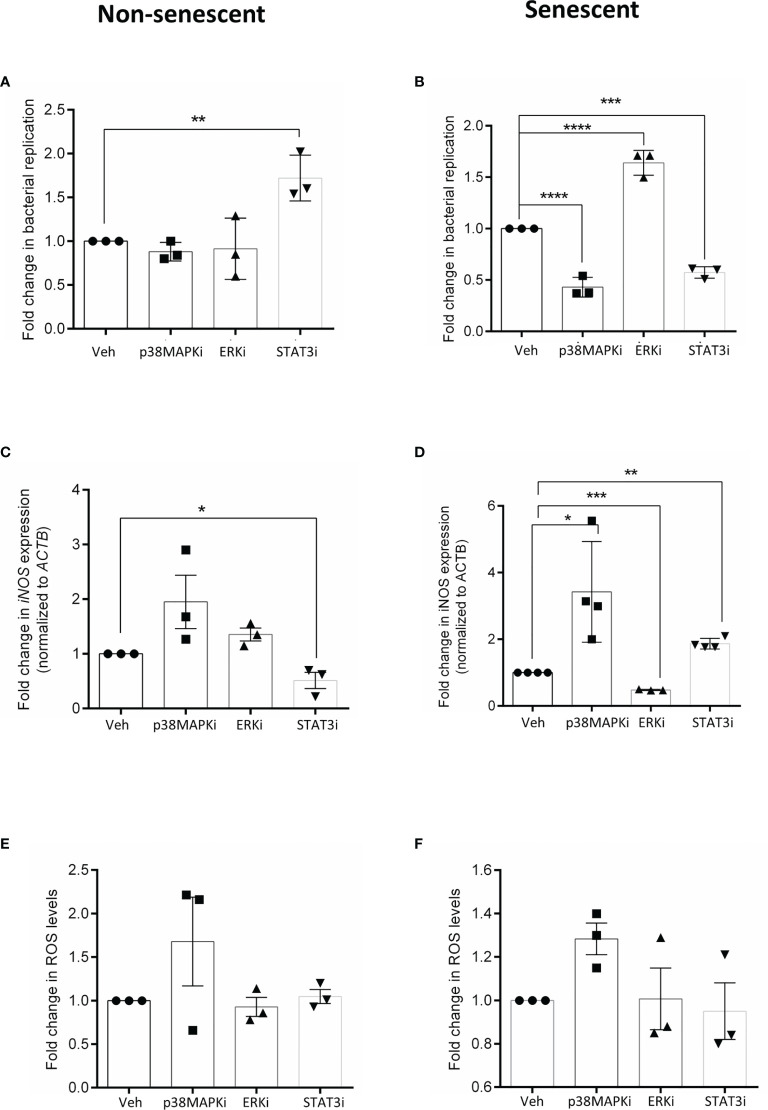
Effect of inhibiting p38 MAPK, ERK and STAT3 kinases on infection in non-senescent (left) and senescent (right) cells and correlates with its effect on cellular iNOS. **(A, B)** Fold change in bacterial replication (relative to vehicle control) at 16h in non-senescent and senescent HeLa cells, respectively after treatment with p38 inhibitor, SB 202190 (p38MAPKi), MEK inhibitor PD184161 (ERKi) and JAK/STAT inhibitor AG490 (STAT3i). **(C, D)** iNOS gene expression analysis by qRTPCR in non-senescent and senescent HeLa cells, respectively. Values were normalized to b-actin and then wrt vehicle-treated cells to determine fold changes. **(E, F)** Determination of ROS levels by DCFDA in non-senescent and senescent HeLa cells, respectively. Fold changes obtained after normalization to levels in vehicle-treated cells. The data represents mean ± SEM from atleast three independent experiments. Statistical significance of differences was analysed by Independent t-test, *P ≤ 0.05, **P ≤ 0.01, ***P ≤ 0.001, ****P ≤ 0.0001. Veh, vehicle DMSO treated cells; p38MAPKi, treated with p38 inhibitor, SB202190; ERKi, treated with MEK inhibitor, PD184161 and STAT3i, treated with JAK/STAT inhibitor, AG490.

The role of these kinases was then probed by infecting cells that were pre-treated for 4h with vehicle (veh) or inhibitor. At 16h post-infection, bacterial replication was estimated to obtain fold changes relative to vehicle-treated cells. In non-senescent HeLa cells, infection significantly increased after inhibiting STAT3 activation (**Figure 2A**) but was otherwise unperturbed in the presence of other inhibitors. On the contrary, when p38MAPK (p38MAPKi) and STAT3 (STAT3i) were inhibited in senescent cells there was a significant decrease in bacterial infection, while ERK inhibition (ERKi) significantly increased intracellular bacterial survival (**Figure 2B**). We also observed the same trends when similar infection experiments were carried out in HepG2 cells (**Supplementary Figures S4A, B**), thus indicating that perturbing these kinases results in a consistent effect on infection and is not cell-line dependent. The contrasting effects of STAT3 inhibition seen between non-senescent and senescent cells highlight that signalling changes occur due to senescence and a particular phenotype, which in this study is the host cell response to bacterial infection, maybe differently regulated by the same molecule.

Several studies have already correlated higher iNOS (Sasaki et al., 1998; Chakravortty and Hensel, 2003) and ROS levels (Fang, 2011; Gogoi et al., 2019) with reduced bacterial infection and further, it has been demonstrated that iNOS plays a pivotal role in determining the infection outcome in senescent cells (Fernandes et al., 2021). Hence, we then compared iNOS transcript levels in vehicle and inhibitor-treated cells. We observed that STAT3 inhibition significantly reduced iNOS transcription (**Figure 2C**) in non-senescent cells, while its transcription significantly increased in p38MAPK and STAT3 inhibited senescent cells (**Figure 2D**). However, ERK inhibition in senescent cells significantly reduced iNOS transcript levels (**Figure 2D**). We also tested the effect of kinase inhibition on cellular ROS, but its levels in inhibitor treated cells were comparable to the vehicle control (**Figures 2E, F**), indicating that iNOS and not ROS primarily drove the effects on bacterial infection.

Together, the data suggest that in senescent cells, ERK activity is necessary for iNOS transcription while p38 MAPK and STAT3 are negative transcriptional regulators of iNOS and hence their further inhibition decreases bacterial infection. In contrast to senescent cells, STAT3 seems to be a positive regulator of iNOS in non-senescent cells, possibly explaining why its inhibition caused an increase in infection. Perturbing the activities of ERK and p38 MAPK did not significantly alter iNOS levels and consequently did not affect bacterial infection in non-senescent cells.

### Cross Talk Between Kinases During Infection in Senescent Cells

Several reports suggest crosstalk between these kinases (Ng et al., 2001; Liu and Hofmann, 2004; Junttila et al., 2008); hence we next sought to investigate if such a regulatory network exists in senescent cells that could influence the cellular anti-bacterial nitrosative response and thereby infection.

**Figure 3:**
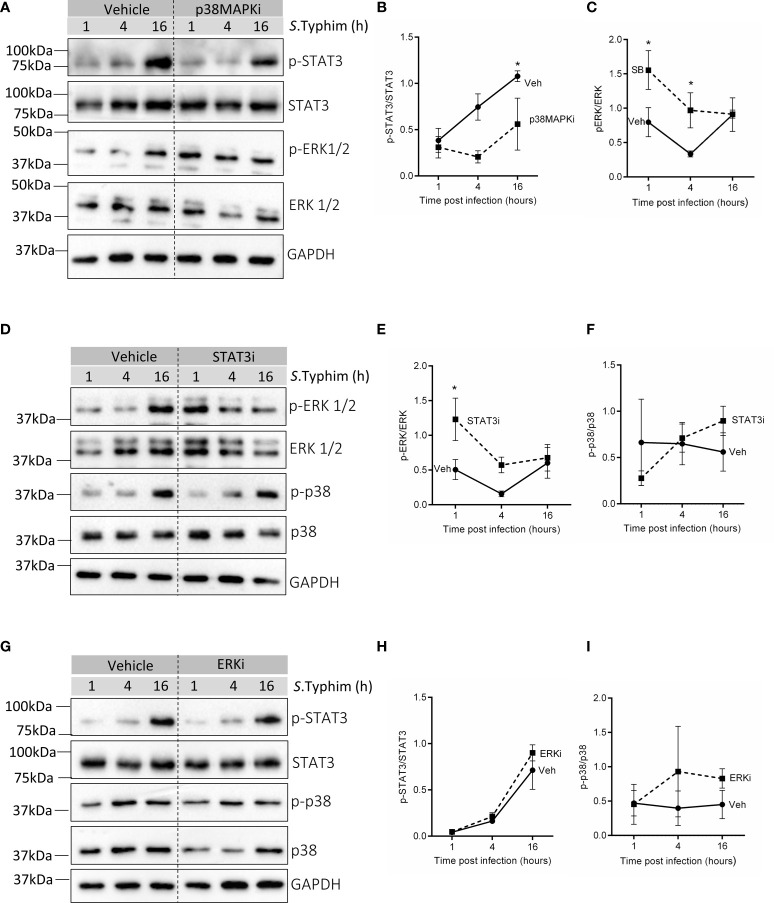
Probing crosstalk of p38 MAPK, ERK and STAT3 in infected senescent cells. **(A)** Western blot for phosphorylated and total levels of STAT3 and ERK1/2 in vehicle or p38MAPKi treated senescent HeLa cells at 1,4 and 16h post-infection. **(B, C)** Quantification of phosphorylated levels of STAT3 and ERK1/2 relative to their total levels, respectively in vehicle or p38MAPKi treated senescent HeLa cells at 1,4 and 16h post-infection. **(D)** Western blot for phosphorylated and total levels of ERK1/2 and p38 MAPK in vehicle or STAT3i treated senescent HeLa cells at 1,4 and 16h post-infection. **(E, F)** Quantification of phosphorylated levels of ERK1/2 and p38 MAPK relative to their total levels, respectively in vehicle or STAT3i treated senescent HeLa cells at 1,4 and 16h post-infection. **(G)** Western blot for phosphorylated and total levels of STAT3 and p38 MAPK in vehicle or ERKi treated senescent HeLa cells at 1,4 and 16h post-infection. **(H, I)** Quantification of phosphorylated levels of STAT3 and p38 MAPK relative to their total levels, respectively in vehicle or ERKi treated senescent HeLa cells at 1,4 and 16h post-infection. In all experiments, GAPDH was used as loading control. The data represents mean ± SEM from atleast three independent experiments. Statistical significance of differences was analysed by Two-way ANOVA followed by posthoc Fisher’s LSD test, *P ≤ 0.05.

Interestingly, blocking p38 MAPK (p38MAPKi) activity in senescent HeLa cells resulted in significantly reduced activation of STAT3 at 4h and 16h post-infection (**Figures 3A, B**) and increased phosphorylation of ERK at early time points (1h and 4h) of infection (**Figures 3A, C**). STAT3 inhibition in senescent HeLa cells was also associated with enhanced p-ERK levels at 1h and 4h post-infection (**Figures 3D, E**) with no effect on p-p38 levels (**Figures 3D, F**). These findings suggest that p38 MAPK and STAT3 possibly dampen ERK activation during infection in senescent cells. Since ERK activity is associated with enhanced iNOS transcription (**Figure 2D**), inhibition of p38 MAPK or STAT3 in senescent cells may result in higher iNOS levels, possibly due to elevated ERK activation. Additionally, inhibition of ERK did not alter the levels of p-p38 (**Figures 3G, H**) or p-STAT3 (**Figures 3G, I**), indicating that p38 MAPK and STAT3 regulate ERK but not vice versa. Moreover, this crosstalk was limited only to infection as inhibition of p38MAPK in senescent HeLa cells did not cause a change in ERK or STAT3 (**Supplementary Figures S5A–D**) phosphorylation and STAT3 inhibition did not alter p-ERK levels (**Supplementary Figures S5C, D**) per se.

Further, in senescent HepG2, not only did p38MAPK inhibition decrease p-STAT3 levels (**Supplementary Figures 6A, B**) like in senescent HeLa, but we also observed that inhibiting p38 MAPK (**Supplementary Figures S6A, C**) or STAT3 (**Supplementary Figures S6D, E**) signalling was associated with increased ERK activation. This suggests that the crosstalk between these pathways is likely to be physiologically relevant to infected senescent cells and is not a cell line-specific effect.

**Figure 4:**
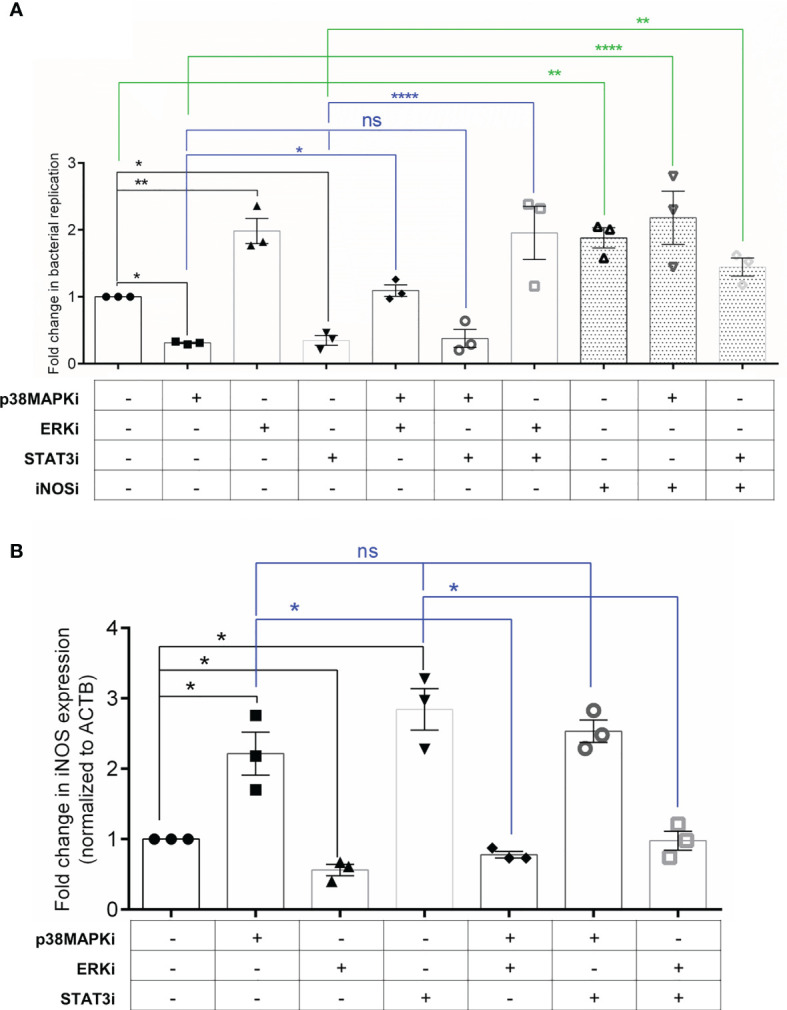
Crosstalk between p38 MAPK, ERK and STAT3 can regulate iNOS levels and infection in senescent cells. **(A)** Fold change in bacterial replication (relative to vehicle control) at 16h in senescent HeLa cells treated with inhibitors as indicated. iNOSi indicates cells treated with iNOS inhibitor, Aminoguanidine. **(B)** iNOS gene expression analysis by qRT-PCR in senescent HeLa cells treated with inhibitors as indicated. Values were normalized to β-actin and then wrt vehicle-treated cells to determine fold changes. The data represents mean ± SEM from atleast three independent experiments. Statistical significance of differences was analysed by Independent t-test, ns, non significant, *P ≤ 0.05, **P ≤ 0.01, ****P ≤ 0.0001.

Finally, to test the functional relevance of this crosstalk network to infection, we performed co-inhibition experiments in both senescent HeLa and HepG2 cells. If blockade of p38 MAPK and STAT3 signalling increases iNOS levels causing reduced intracellular bacterial survival, then inhibition of iNOS in p38MAPKi and STAT3i senescent cells should rescue this effect on infection. We observed that indeed co-inhibiton with Aminoguanidine (AMG), a specific inhibitor of iNOS (represented as iNOSi in the graphs) resulted in an increase in bacterial infection compared to p38MAPKi or STAT3i senescent cells. Further, if the effect of p38 MAPK and STAT3 inhibition on iNOS is *via* enhanced p-ERK levels, then inhibiting p-ERK activation in p38MAPKi or STAT3i senescent cells should abrogate the increase in iNOS levels and its resultant effect on bacterial infection. Co-inhibited senescent Hela cells showed that ERK inhibition could reduce iNOS levels (**Figure 4B**) and further rescue bacterial survival in p38MAPKi or STAT3i cells (**Figure 4A**). We also validated these findings in senescent HepG2 cells and found a similar effect of co-inhibition on bacterial infection (**Supplementary Figure S7**). Additionally, the absence of an additive effect of p38MAPK and STAT3 inhibition on iNOS transcription and bacterial infection, along with our previous observation that inhibiting p38 MAPK decreased STAT3 activation but not vice versa (**Figures 3C, F**) re-enforces the possibility of a unidirectional positive regulatory loop between p38 MAPK and STAT3 in infected senescent cells.

## Discussion

The accumulation of senescent cells during aging is a major driver of frailty in the elderly (Fedarko, 2011; Childs et al., 2015). Besides this, patients suffering from Chronic Obstructive Pulmonary Disorders (COPD) and cigarette smokers also show a higher prevalence of senescent cells in the lungs (Nyunoya et al., 2006; Vij et al., 2018; Parikh et al., 2019). So far, the focus of most studies has been to understand how intrinsic and secretome changes characteristic of senescent cells exacerbates geriatric and inflammation associated pathologies (Jeon et al., 2018; Zhang et al., 2019; Nehme et al., 2020; Camell et al., 2021) but very little is known about the alteration in the signalling landscape of senescent cells.

In this study, we use an infection model to highlight that key signalling proteins like ERK, p38 MAPK and STAT3 are activated differently in senescent cells compared to their non-senescent counterparts. Using specific molecular inhibitors, we also elucidated their contrasting function in regulating infection *via* cellular iNOS levels, a critical anti-microbial response (Henard and Vázquez-Torres, 2011; Gogoi et al., 2019) and has been previously demonstrated to increase in senescent cells (Fernandes et al., 2021). Interestingly, the inhibition of STAT3 led to a decrease in iNOS levels in non-senescent cells, which has also been previously reported (Lo et al., 2005; Ziesché et al., 2007). However, its inhibition in senescent cells caused an increase in iNOS levels, providing evidence that STAT3 regulates the same response differently in non-senescent and senescent cells. Further, many groups have demonstrated that the continuous activation of ERK can induce cellular senescence (Deschênes-Simard et al., 2013; Zou et al., 2019) but very few studies have focused on its role thereafter. In this study, we have identified a novel role of ERK in senescent cells, specifically, its ability to regulate iNOS transcription. Furthermore, our co-inhibition experiments also revealed that both STAT3 and p38 MAPK were able to alter iNOS levels by decreasing ERK activation in senescent cells, suggesting its pivotal in the transcriptional regulation of iNOS.

**Figure 5:**
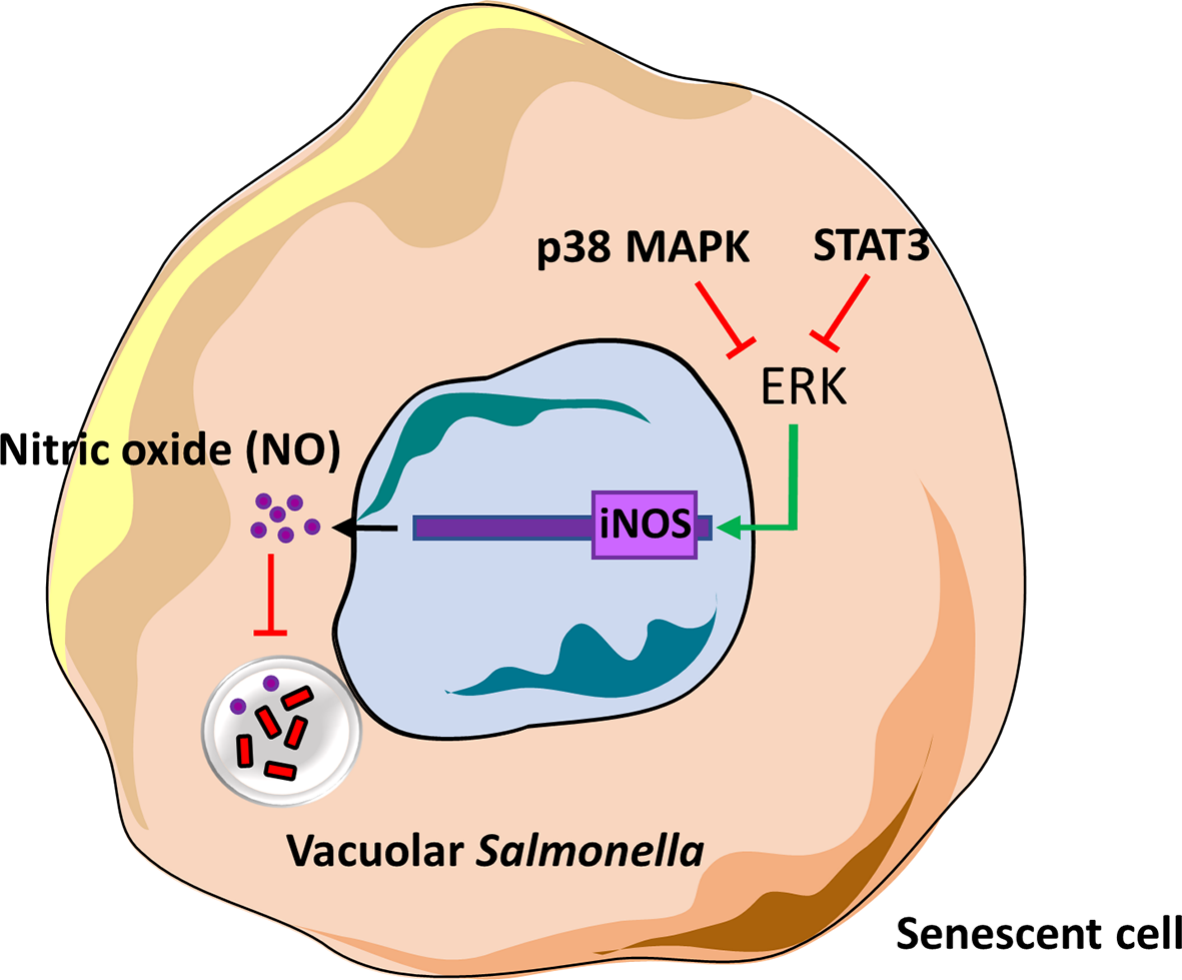
Model of iNOS regulation by p38 MAPK, ERK and STAT3 in senescent cells.

In summary, we have delineated one of the roles of p38 MAPK, ERK and STAT3 in senescent cells and based on our findings, depict the presence of a novel cross-regulatory mechanism between these kinases (**Figure 5**). These kinases may have been previously studied in the context of senescence and DNA damage signalling, cell survival or inflammation but for the first time we demonstrate their role in regulating infection in senescent cells.

## Data Availability Statement

The original contributions presented in the study are included in the article/**Supplementary Material**. Further inquiries can be directed to the corresponding author.

## Author Contributions

SF designed the study, performed the experiments, analysed the data and wrote the manuscript. DS conceived the study, analysed the data and wrote the manuscript. All authors contributed to the article and approved the submitted version.

## Funding

This work was supported by funding from the Infosys Foundation to IISc, Department of Biotechnology, India (Grant No. DBT/PR12121/BRB/10/1332/2014) to DS. The study is also supported by the DBT partnership program to the Indian Institute of Science (DBT/PR27952-INF/22/212/2018). Equipment support by DST– Funds for Infrastructure in Science and Technology program (SR/FST/LSII-036/2016).

## Conflict of Interest

The authors declare that the research was conducted in the absence of any commercial or financial relationships that could be construed as a potential conflict of interest.

## Publisher’s Note

All claims expressed in this article are solely those of the authors and do not necessarily represent those of their affiliated organizations, or those of the publisher, the editors and the reviewers. Any product that may be evaluated in this article, or claim that may be made by its manufacturer, is not guaranteed or endorsed by the publisher.
