# Supplementary material for: The ERK-p38MAPK-STAT3 Signalling Axis Regulates iNOS Expression and Salmonella Infection in Senescent Cells
Source: Front Cell Infect Microbiol. 2021 Oct 22;11:744013. doi: 10.3389/fcimb.2021.744013 (PMC8569389; doi:10.3389/fcimb.2021.744013)
Supplement: Supplementary file 1 [file DataSheet_1.pdf]

Supplementary Fig S1:

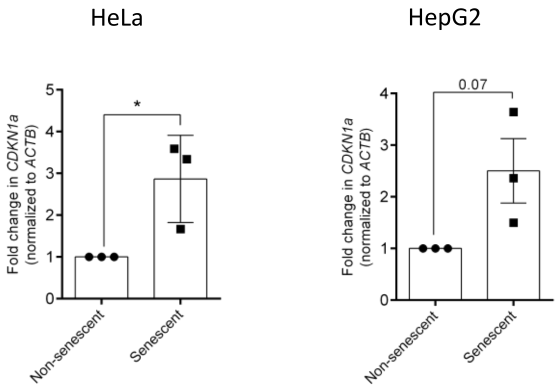

qRT-PCR analysis for *CDKN1a* gene expression. Expression was normalised to β-actin and fold change has been represented wrt non-senescent cells. The data represents mean ± SEM from three independent experiments. Statistical significance of differences was analysed by Student’s t-test, $^{*}P \leq 0.05$.

Supplementary Fig S2:

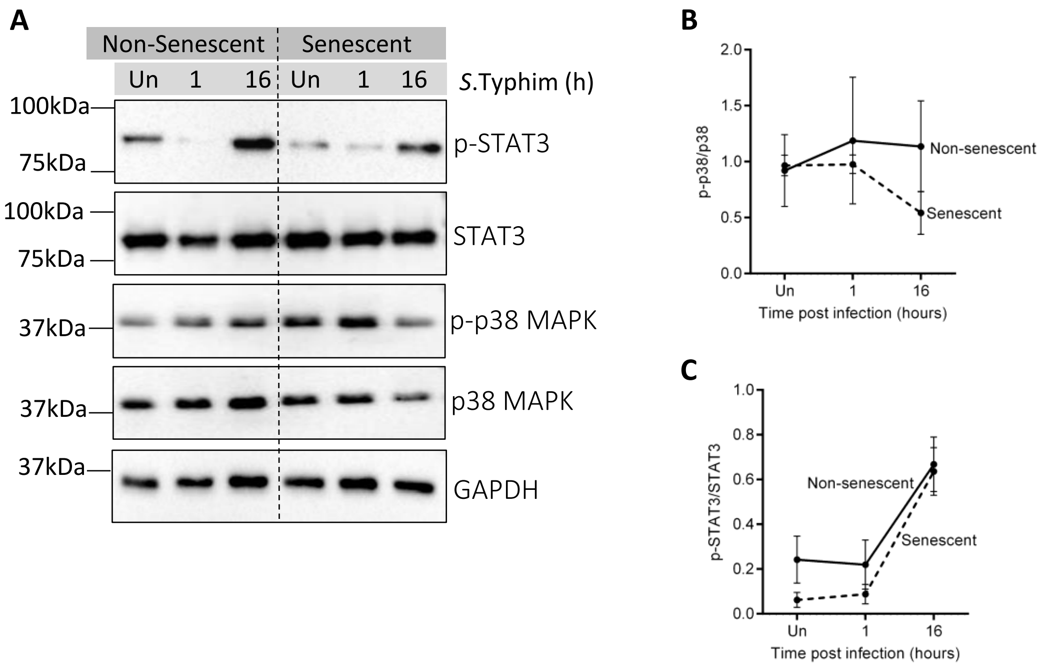

Kinetics of STAT3 and p38 activation in infected non-senescent and senescent HepG2 cells. A: Western blot for phosphorylated and total levels of STAT3 and p38 MAPK in uninfected and infected (1h and 16h post infection) non-senescent and senescent cells. GAPDH used as loading control. **B and C:** Quantification of phosphorylated levels of p38 MAPK and STAT3 relative to their total levels in uninfected and infected non-senescent and senescent cells, respectively. The data represents mean ± SEM from atleast three independent experiments.

Supplementary Fig S3:

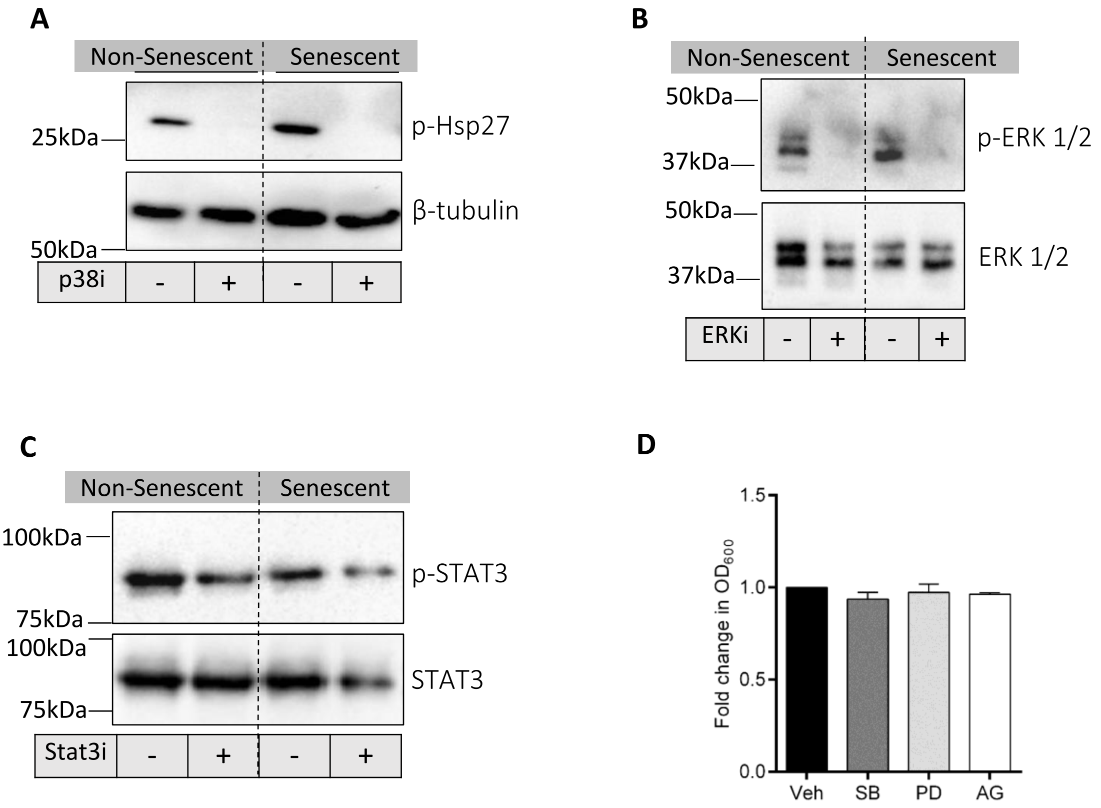

Molecular inhibitors used decrease the activity of their target molecules in non-senescent and senescent HeLa cells without compromising bacterial viability. A: SB inhibition of activated p38 MAPK demonstrated by abrogated activation of its substrate Hsp27. **B:** PD inhibition of MEK resulting in reduced ERK phosphorylation **C:** AG inhibition of JAK1/2 resulting in reduced STAT3 phosphorylation. **D:** Viability (measured in terms of OD_{600}) of *S.*Typhimurium growing in the presence of veh/inhibitors (10µM) for 24h.

Supplementary Fig S4:

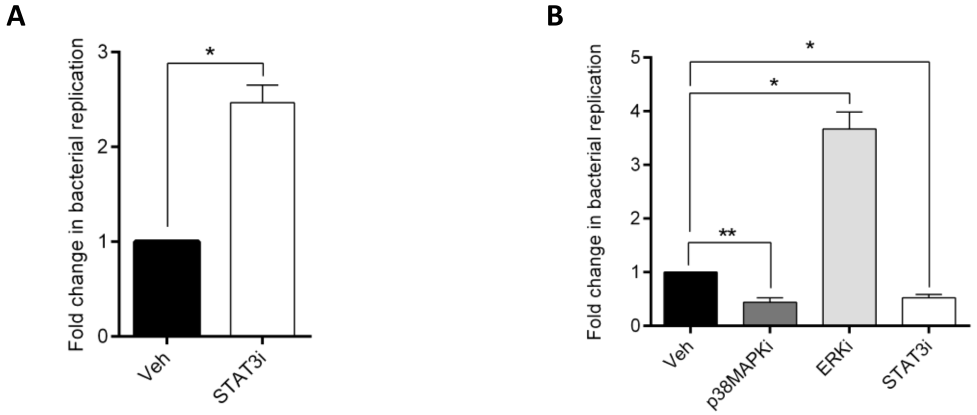

Effect of kinase inhibition on infection in HepG2 cells. A: Fold change in bacterial replication at 16h in STAT3i non-senescent HepG2 cells relative to vehicle (veh) treated cells. **B:** Fold change in bacterial replication (relative to vehicle control) at 16h in senescent cells after treatment with SB 202190 (p38MAPKi), PD184161 (ERKi) and AG490 (STAT3i). The data represents mean ± SEM from atleast three independent experiments. Statistical significance of differences was analysed by Independent t-test, *P ≤ 0.05, **P ≤ 0.01.

Supplementary Fig S5:

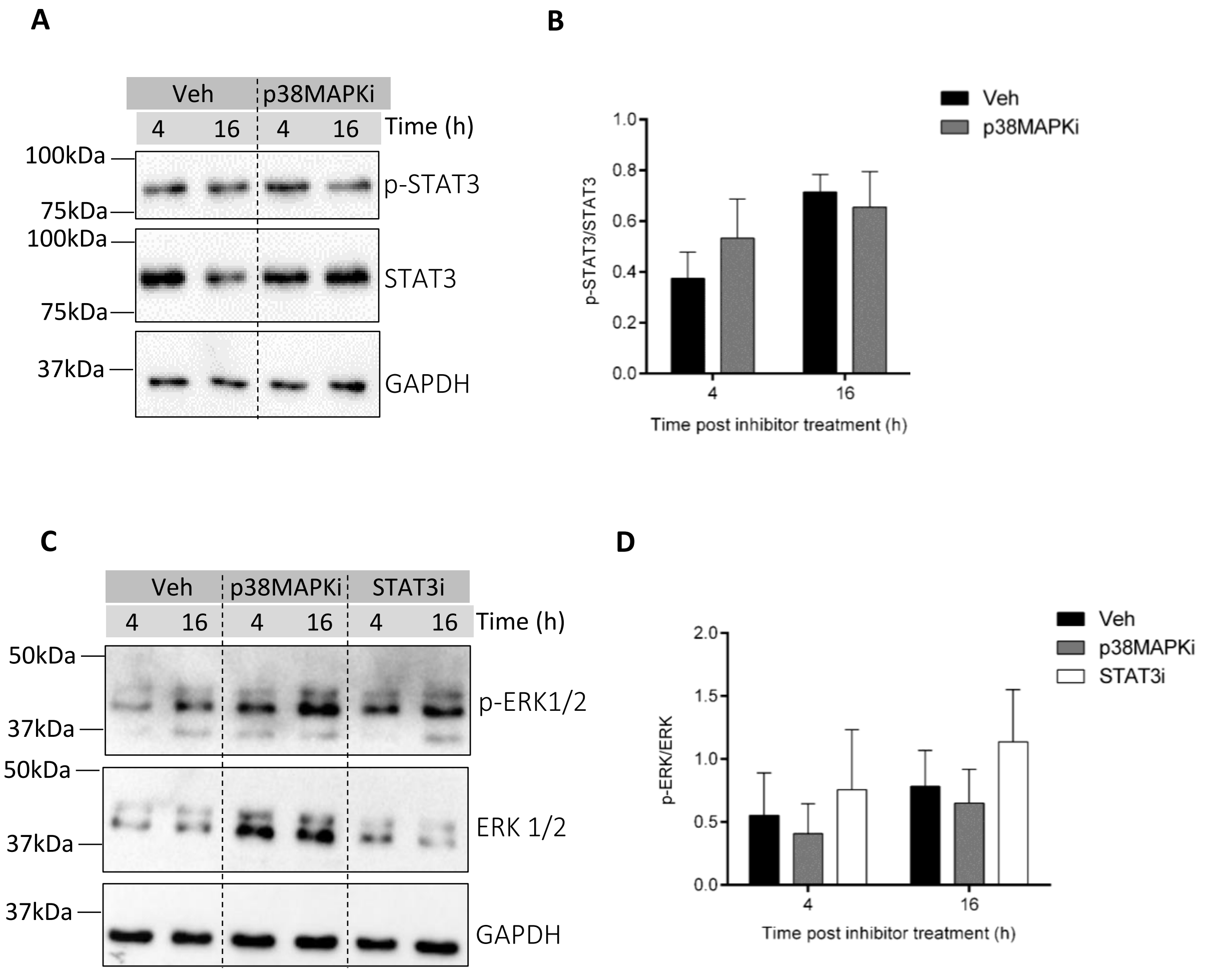

Cross talk of p38 with STAT3/ERK and STAT3 with ERK is specific to infected senescent cells. A: Western blot for STAT3 activation in senescent HeLa at 4h and 16h post vehicle (veh) treatment and after p38MAPK inhibition **B:** Quantification of phosphorylated levels of STAT3 to its total levels in veh/p38MAPKi senescent HeLa. **C:** ERK activation in p38MAPKi or STAT3i senescent HeLa. **D:** Quantification of phosphorylated levels of ERK relative to its total levels in veh/p38MAPKi/STAT3i senescent HeLa. GAPDH used as loading control. The data represents mean ± SEM from atleast three independent experiments.

Supplementary Fig S6:

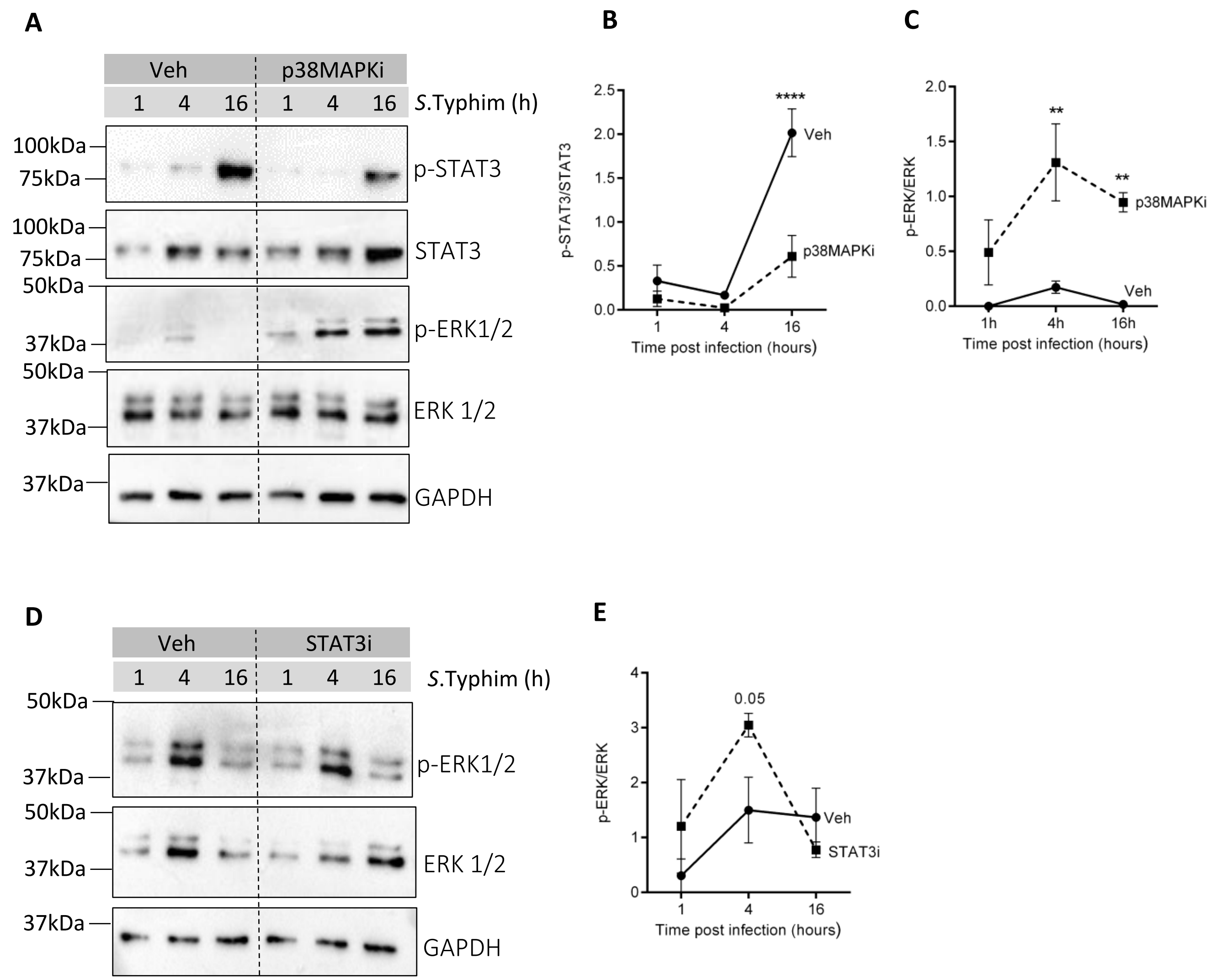

Cross talk between p38 MAPK, ERK and STAT3 is also observed in infected senescent HepG2 cells. A: Western blot for phosphorylated and total levels of STAT3 and ERK1/2 in vehicle or p38MAPKi senescent cells at 1,4 and 16h post infection. **B and C:** Quantification of phosphorylated levels of STAT3 and ERK1/2 relative to their total levels, respectively in vehicle or p38MAPKi senescent cells at 1,4 and 16h post infection. **D:** Western blot for phosphorylated and total levels of ERK1/2 in vehicle or STAT3i senescent cells at 1,4 and 16h post infection. **E:** Quantification of phosphorylated levels of ERK1/2 relative to its total levels, respectively in vehicle or STAT3i senescent cells at 1,4 and 16h post infection. The data represents mean ± SEM from atleast three independent experiments. Statistical significance of differences was analysed by Two-way ANOVA followed by posthoc Fisher's LSD test,$^{**}P \leq 0.01$, $^{****} P \leq 0.0001$.

Supplementary Fig S7:

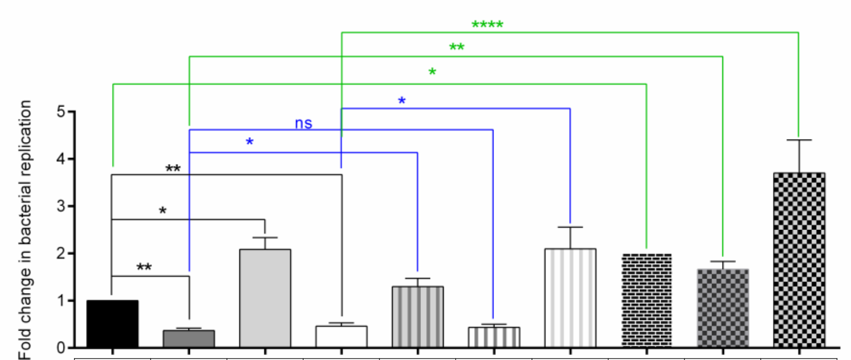

**p38MAPKi**	-	+	-	-	+	+	-	-	+	-
**ERKi**	-	-	+	-	+	-	+	-	-	-
**STAT3i**	-	-	-	+	-	+	+	-	-	+
**iNOSi**	-	-	-	-	-	-	-	+	+	+

Crosstalk between p38 MAPK, ERK and STAT3 can regulate infection in senescent HepG2 cells. Fold change in bacterial replication (relative to vehicle control) at 16h in senescent HepG2 cells treated with inhibitors as indicated. The data represents mean ± SEM from atleast three independent experiments. Statistical significance of differences was analysed by Independent t-test, ns-non significant, *P ≤ 0.05, **P ≤ 0.01, **** ≤ 0.0001.

Table S1: Primers used in the study

Primer Name	Primer sequence (5' → 3')
ACTB (β-actin)f	ccaaccgcgagaagatgac
ACTB (β-actin)r	cagaggcgtacagggatagc
iNOS f	cagcgggatgactttccaa
iNOS r	aggcaagatttggacctgca
CDKN1a f	ggaagaccatgtggacctgt
CDKN1a r	tagggcttcctcttggagaa

Table S2: Antibodies used in the study

Antibody	Dilution used	Catalogue number
Phospho p38 MAPK	1:1000	4511
p38 MAPK	1:1000	9212
Phospho ERK	1:1000	9106
ERK	1:1000	4695
Phospho STAT3	1:2000	9145
STAT3	1:2000	4904
Phospho Hsp27	1:1000	9709
GAPDH	1:1000	2118
β-tubulin	1:1000	2146
